# X-Linked miRNAs Associated with Gender Differences in Rheumatoid Arthritis

**DOI:** 10.3390/ijms17111852

**Published:** 2016-11-08

**Authors:** Olfa Khalifa, Yves-Marie Pers, Rosanna Ferreira, Audrey Sénéchal, Christian Jorgensen, Florence Apparailly, Isabelle Duroux-Richard

**Affiliations:** 1 Inserm, U1183, Institute for Regenerative Medicine and Biotherapies, CHU Saint Eloi, 80 Avenue Augustin Fliche, 34295 Montpellier, France; olfa.khalifa@inserm.fr (O.K.); ympers2000@yahoo.fr (Y.-M.P.); christian.jorgensen@inserm.fr (C.J.); florence.apparailly@inserm.fr (F.A.); 2 University of Montpellier, Boulevard Henri IV, 34090 Montpellier, France; 3 Clinical Department for Osteoarticular Diseases and Biotherapy, University Hospital Lapeyronie, 34295 Montpellier, France; rosannaferreiralopez@gmail.com; 4 Inserm, U1051, Institute for Neurosciences Montpellier, CHU Saint Eloi, 80 Avenue Augustin Fliche, 34295 Montpellier, France; audrey.senechal@inserm.fr

**Keywords:** rheumatoid arthritis, miRNA, gender, X-chromosome, FoxP3

## Abstract

Rheumatoid arthritis (RA) is an autoimmune disease that predominantly affects women. MicroRNAs have emerged as crucial regulators of the immune system, whose expression is deregulated in RA. We aimed at quantifying the expression level of 14 miRNAs located on the X chromosome and at identifying whether differences are associated with disease and/or sex. A case–control study of 21 RA patients and 22 age- and sex-matched healthy controls was performed on peripheral blood mononuclear cells. The expression level of five miRNAs (miR-221, miR-222, miR-532, miR-106a, and miR-98) was significantly different between RA and controls when stratifying by sex, and the expression level of four miRNAs (miR-222, miR-532, miR-98, and miR-92a) was significantly different between RA females and males. The expression quantitative trait loci (eQTL) analysis revealed a significant gender effect of the FoxP3 promoter polymorphism rs3761548A/C on miR-221, miR-222 and miR-532 expression levels, and of the FoxP3 polymorphism rs2232365A/G on miR-221 expression levels in PBMC of RA patients. These data further support the involvement of the X chromosome in RA susceptibility. X-linked miRNAs, in the context of sex differences, might provide novel insight into new molecular mechanisms and potential therapeutic targets in RA for disease treatment and prevention.

## 1. Introduction

The majority of autoimmune diseases predominate in females. In some diseases such as rheumatoid arthritis (RA), females are three times more affected than men. RA is a chronic autoimmune disease affecting 0.5%–1% of the population worldwide. The disease has heterogeneous features such as joint inflammation, synovial hyperplasia, and joint destruction [1]. The etiology of this complex disease is still poorly understood but is considered as a result of an interaction between susceptibility genes and environmental factors including geography, climate, endemic microbes, and socio-culture practices, like smoking, lifestyle, and dietary habits [2]. Many hypotheses for female predisposition have been investigated. First, hormones may play a role as hormonal changes during menopause or pregnancy impact RA severity. Estrogen treatment results in an increase in Th17 cells in lymph nodes during the early phase of development in experimental autoimmune arthritis [3]. Many studies focusing on the X chromosome-linked genes showed that differences in immune response and autoimmunity affecting both the innate and adaptive immune response contribute to differences between males and females in the pathogenesis of RA [4]. The contribution of epigenetic modifications, microchimerism and skewed X-chromosome inactivation to RA female predominance has also been studied [5,6,7].

Among abnormal epigenetic modifications that have been evidenced in RA, microRNAs (miRNAs) are of interest. miRNAs are small non-coding RNA molecules that post transcriptionally regulate the expression of protein-encoding genes. They control gene expression through various mechanisms such as translational repression and RNA degradation [8]. As protein-encoding genes, miRNA-encoding genes can be affected by nucleotide single polymorphisms (SNPs) that could affect their expression levels in disease. For example, the rs3746444 located in pre-miR-499 is associated with clinical markers of inflammation and disease severity in RA patients [9,10]. On the other hand, “silent” mutation in protein-encoding genes can alter miRNA function, as a synonymous variant in the IRGM gene impairs the binding of miR-196 and increases the risk for Crohn’s disease [11]. The lasted version of miRNA databases (miRBase21) records 1881 human miRNAs precursors and 2588 mature miRNAs in the human genome. The human X chromosome is highly enriched in miRNAs as compared to the Y-chromosome, being second just after the chromosome 1 that encodes 134 miRNAs, with 116 X-linked miRNAs against only two Y-linked miRNAs. The most studied X chromosome-linked miRNA in autoimmune disorders is miR-223. In RA, deregulated miR-223 expression has been detected in various cell types. miR-223 is upregulated in CD3+ T-lymphocytes from peripheral blood of RA patients [12]. It plays a critical role in inflammation by controlling the differentiation and maturation process of various immune cells, including osteoclast and granulocytic differentiation of myeloid precursors [13,14]. However, there are no studies exploring differential regulation of miR-223 in males versus females in RA, and studying the role of other miRNAs in sex-biased RA.

## 2. Results

### 2.1. Characteristics of Patients

The characteristics of the 21 seropositive RA patients and 22 healthy donors are shown in Table 1.

All patients were recruited from the Clinical department of Osteoarticular diseases and Biotherapy at the University Hospital Lapeyronie, Montpellier, Southeastern of France. All RA patients presented non-inflammatory disease as assessed by a low disease activity score with over 76% of patients in remission (DAS 28 < 2.6), without other known pathology. The mean RA duration was 17.3 ± 11.3 years, 100% were ACPA (anti-citrullinated peptide antibodies) and rheumatoid factor (RF) positive. All patients were under treatment and drug types are detailed in Table 1. The mean dose of prednisolone and methotrexate were 22.5 ± 12 mg/daily and 14 ± 4 mg/week, respectively. The average age was 60 ± 12 years for RA patients and 54.7 ± 6.4 years in controls, and smoking status did not differ significantly between case and control groups.

### 2.2. miR-146a and miR-223 Expression Levels Do Not Discriminate RA Patients with Low Disease Activity Score

**Figure 1:**
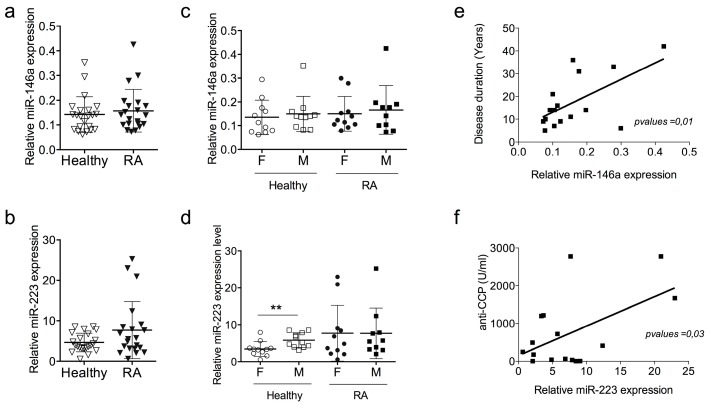
Expression levels of miR-146a and miR-223 in PBMCs of RA patients with low disease activity score. PBMCs were isolated from the blood of patients with rheumatoid arthritis (RA) or healthy controls and the expression levels of miR-146a and miR-223 were quantified using real-time RT-PCR. For normalization, the endogenous RNU48 was used. (**a**,**b**) Expression levels of miR-146a and miR-223 in RA patients and healthy donors. Results are expressed as mean ± SD of individual sample of 21 RA patients and 22 healthy subjects; (**c**,**d**) Patients were divided according to their gender in each group (RA and HC). Results are expressed as mean ± SD of individual sample of 10 RA females, 11 RA males, 11 healthy females and 10 healthy males, ** *p* < 0.01, Mann–Whitney test; (**e**,**f**) Correlations between miRNA expression levels and clinical characteristics of the cohort, miR146a and miR-223 expression level between disease duration (**e**) and anti-cyclic citrullinated peptide (Anti-CCP) antibodies titers (**f**), respectively.

As prototypic miRNAs are deregulated in RA, we compared the expression levels of one miRNA encoded on X chromosome (miR-223) and of one miRNA encoded on a non-sex chromosome (miR-146a). Using RT-qPCR to quantify the expression level of both miRNAs in our prospective cohort, we found no difference between healthy subjects and patients with non-active RA, although a tendency to overexpression of miR-223 was observed in RA patients (Figure 1a,d). To assess whether sex differences affect miRNA expression levels, we stratified RA patients and healthy subjects according to their sex. No difference was observed for miR-146a between females and males, both in the RA group and in healthy subjects (Figure 1b). However, the expression level of miR-223 was significantly higher in males than in females in the group of healthy subjects, and such a sex-bias was eliminated in disease (Figure 1e). No correlation was found between miR-146a or miR-223 expression and biological parameters of RA patients such as DAS28, CRP, anti-CCP and RF (Figure S1). Significant correlations (*p*-values < 0.01, <0.03) were observed for RA patients between miR-146a expression and disease duration (Figure 1e) and between miR-223 expression and ACPA concentrations (Figure 1f), which highlights the role of such miRNA in RA pathology, regardless of disease activity.

### 2.3. Sexual Dimorphism of miRNA Expression in RA

**Figure 2:**
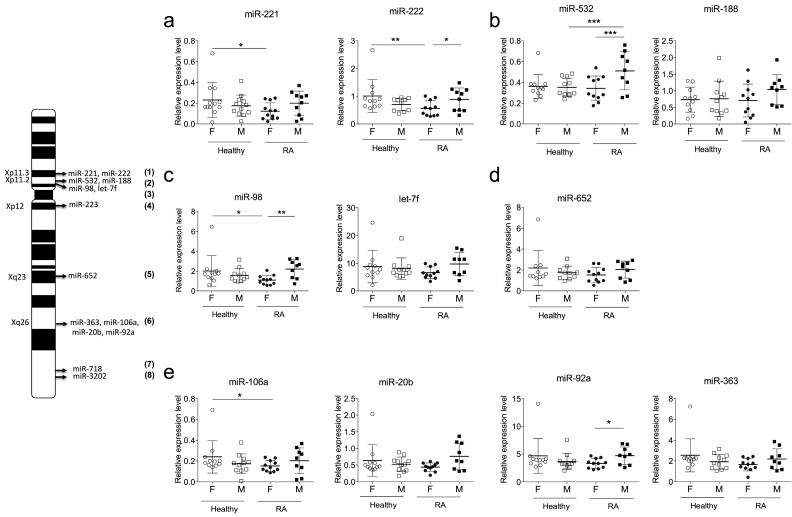
Sexual dimorphism of miRNA expression in RA. Representative scheme of the 11 selected X-linked: miR221/222 (**1**); miR532/188 (**2**); miR-98/let-7f (**3**); miR-223 (**4**); miR-652 (**5**); miR-363/106a/20b/92a (**6**); miR-718 (**7**) and miR-3202 (**8**). (**a**–**e**) Expression levels of 11 X-linked miRNAs: miR-221 and miR-222 (**a**); miR-532 (**b**); miR-98 (**c**); miR-223 and miR-652 (**d**); and miR-106a, miR-20d, miR-363 and miR-92a (**e**) were detected on PBMCs isolated from the blood of RA patients and healthy controls using RT-PCR. For normalization, the endogenous RNU48 was used. In each group, subjects were divided according to their gender. miR-718 and miR-3202 are not detectable. Results are expressed as mean ± SD of 21 RA patients and 21 healthy subjects, * *p* < 0.05, ** *p* < 0.01, *** *p* < 0.001, Mann–Whitney test.

The human X chromosome encodes 116 miRNAs, and more than 53% of them are common with the mouse genome. In humans, 28% and 16% of X-linked miRNAs are implicated or described in cancer or immunity, respectively. Among these miRNAs, a little less than 20% are deregulated in autoimmune diseases or located near SNPs associated with autoimmune disorders such as RA or Systemic lupus erythematosus (SLE). These miRNAs are listed in Table 2 and their respective positions on the X chromosome are shown in a schematic representation (Figure 2).

Each miRNA listed in Table 2 was quantified with real-time PCR. Absence of significant correlation was observed between X-linked miRNAs and most of the biological parameters of RA patients. A significant correlation was only found between the erythrocyte sedimentation rate and the expression level of miR-221, miR-222, Let-7f-2 and miR-652 (Table 3). Finally, the therapy used in RA patients had no effect on the expression level of X-linked miRNAs (Figure S2).

Results were divided according to the sex and disease status. We identified five X-linked miRNAs with significantly different expressions in RA according to the sex. miR-221 (*p*-value = 0.02), miR-222 (*p*-value = 0.01), miR-98 (*p*-value = 0.02) and miR-106a (*p*-value = 0.04) were down deregulated in PBMC of RA females versus control females (Figure 2a,c,f), while miR-532 was up regulated (*p*-value = 0.001) in PBMC of RA males versus healthy males.

In RA patients, four X-linked miRNAs had different expressions depending on the sex: miR-222 (*p*-value = 0.046), miR-532 (*p*-value = 0.001), miR-98 (*p*-value = 0.007), and miR-92a (*p*-value = 0.04) were significantly down deregulated in PBMC of female versus male RA patients (Figure 2a–c,f). Without stratification by sex, no significant difference in the expression levels of X-linked miRNAs was observed between RA and healthy donors (Figure S3).

### 2.4. SNPs in FOXP3 Might Influence miR-221, miR-222 and miR-532 Levels of Expression

**Figure 3:**
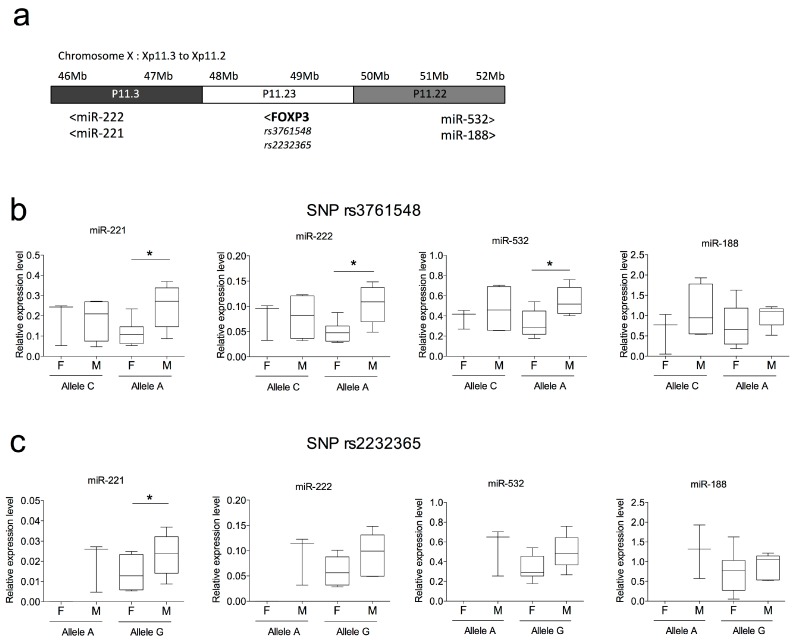
Analysis of transcriptional regulatory relationships between miRNA expression levels and two *FOXP3* variants associated with RA susceptibility. (**a**) Scheme representing the locus Xp11.3 to Xp11.2 of the human X chromosome shows the distance and position of two miRNA clusters (miR-222/miR-221 and miR-532/miR-188) located near two *FOXP3* SNPs (rs3761548 and rs2232365) associated with RA; (**b**,**c**) Analysis of the transcriptional regulatory relationships between miR-221, miR-222, miR-532 and miR-188 expression levels and *FOXP3* rs3761548A/C (**b**) and rs2232365A/G (**c**) variants associated with RA. Box plots represent individual sample of 11 RA female and 10 RA male PBMCs. * *p* < 0.05, Mann–Whitney test.

Analysis of expression quantitative trait loci (eQTL) provides a means for detecting transcriptional regulatory relationships between gene expression levels and DNA variants. Among miRNAs listed in Table 2, two clusters are located near polymorphisms associated with RA susceptibility: the miR221/222 and miR532/188 clusters are located near two *FOXP3* polymorphisms (rs3761548 and rs2232365). Figure 3a shows schematically the relative position of both miRNA clusters and distances from both *FOXP3* SNPs.

We thus investigated whether gene expression levels of miR-221, miR-222, miR-532 and miR-188 were affected by both *FOXP3* polymorphisms in RA patients. Patients were divided into two groups according to their genotypes: the wild type homozygous genotype (CC for rs3761548 and AA for rs2232365), and the genotype carrying the variant, allele A for rs3761548 and allele G for rs2232365. The miR-eQTL analysis was performed with and without sex stratification. The results revealed a significant relationship between *FOXP3* promoter polymorphism rs3761548A/C and miR-221, miR-222 and miR-532 expression levels in PBMC of RA patients only when stratifying by sex (Figure 3b and Figure S4). Regarding the *FOXP3* polymorphism rs2232365A/G, only miR-221 showed a significant difference (*p*-value = 0.03) between RA males and females in variant allele G (Figure 3c). Notably, 100% of the genotyped RA females presented the *FOXP3* polymorphism rs2232365A/G, while only 60% of the RA males did. No differences between combined and separated effect of *FOXP3* SNPs (rs3761548 and rs223236) for miR-222 and miR-222 were observed (data not shown). No differences in the levels of miR-221/222, miR-532 and miR-188 were observed in healthy controls of different genotypes in PBMCs (data not shown).

## 3. Discussion

In humans, X chromosome-associated mechanisms can have beneficial effects for females such as longer life expectancies or best survival outcome from shock episodes caused by sepsis, injury or trauma-hemorrhage as compared with males [15]. However, such mechanisms can also increase susceptibility to develop autoimmune disorders such as Rheumatoid arthritis (RA), Sjögren’s syndrome or systemic lupus erythematosus (SLE) [16,17]. In RA, females are three times more exposed than males [18]. Many factors can play an important role in sex bias in autoimmune diseases such as genetic, hormonal and life style factors. Differences between females and males, susceptibility to disease and treatment outcome result from direct genetic differences [19,20]. Within X chromosome, many genes are directly or indirectly involved in the immune response including FOXP3, IRAK1, and MeCP2. More recently, epigenetic factors have been involved, among which miRNAs. miRNA dysregulation is linked to auto-immune pathologies, including RA, and seems to contribute to the molecular mechanisms involved in the immune response [21,22]. Interestingly, 74% of the 116 X-linked miRNAs are clustered: miR-221/222, miR532/188, miR-98/Let7f, and miR-363/106a/20b/92a. A number of X-linked miRNAs are intronic in known protein-coding genes, and thus generally believed to be co-transcribed and co-expressed. This would be the case for cluster miR-532/188 within CLCN5 (chloride voltage-gated channel 5); cluster miR-98/let7-f within HUWE1 (HECT, UBA and WWE Domain Containing 1, E3 Ubiquitin Protein Ligase); miR-718 within IRAK1 (Interleukin-1 Receptor-Associated Kinase 1); miR-652 within TMEM164 (Transmembrane Protein 164); and miR-3202 within TMEM187 (Transmembrane Protein 187). Intergenic miRNAs are independent transcription units. Only one study reports that 149 miRNAs were differentially expressed between males and females mouse neonatal brain [23]. In humans, most of the miRNA-based studies however ignore the gender context, which can lead to biased results.

In the present study, we have investigated variations in the expression levels of 14 X-linked miRNAs in PBMCs of RA females and males as compared with healthy male and female donors. We have observed that six miRNAs (miR-221, miR-222, miR-98, miR-532, miR-106a and miR-92a) display significant sexual dimorphisms. Interestingly, all six of these miRNAs have already been described either in RA or in inflammation. The expression of the miR-221/miR-222 cluster is significantly upregulated in synovial fibroblasts (FLS) isolated from the human TNF transgenic mouse model of RA [24]. miR-221 is implicated in RA pathogenesis since a downregulation of this miRNA inhibits the expression of pro-inflammatory cytokines and chemokines, suppresses RA-FLS cell migration and invasion, and induces cell apoptosis [25]. Furthermore, miR-222 is involved in cartilage destruction by targeting HDAC-4 and regulating MMP-13 level [26]. miR-98 is upregulated in OA cartilage, and functional pathway analysis of the predicted gene targets suggest that this miRNA plays an inflammatory role by controlling the expression of IL-1β, TNF-α, and MMP-13 [27]. Differences between studies underscore the importance of the cellular context. miR-532 is involved in the inflammatory response of monocytes to lipopolysaccharide (LPS) [28]. In SLE, miR-106a is significantly decreased in the plasma of patients with SLE compared with healthy donors [29]. Moreover, miR-106a regulates IL-10 expression, which is in turn transcriptionally regulated by Egr1 and Sp1 [30]. Finally, following stimulation with TLR2/4 ligands, miR-92a decreases in macrophages and controls inflammatory response by targeting MKK4/JNK/c-Jun pathway [31].

Surprisingly, we did not find differences in expression levels of miR-146a and miR-223 in RA patients as compared with healthy subjects. These miRNAs are indeed described as highly upregulated in RA, playing important roles in the negative regulation of inflammatory innate immune responses [32] and being correlated with disease activity [33,34]. Additionally, it has been shown that low expression levels of miR-146a, which are comparable to those of PBMCs isolated from healthy donors, are correlated with inactive RA disease [33]. miR-223 expression level is correlated with ACPA concentration, suggesting again specificity regulation of these miRNAs in inflammatory condition, furthermore miR-223 is overexpressed in serum/plasma from RA patients compared with healthy donors [35] and its expression has been described as decreased at three and 12 months after treatment initiation [36]. The similarity of miR-146a and miR-223 expression levels between healthy subjects and RA patients observed in the present study might thus be due to the cohort used, which contains predominantly RA patients with remission or at least low disease activity (DAS28 < 2.6). However, we found that miR-146a expression was correlated with disease duration, suggesting that its expression is higher in patients with chronic inflammatory activity than in patients in remission.

Blood is a heterogeneous tissue where differences in cell type composition may confound discovery of differences in miRNA expression that we attributed to sex and only reflect blood enrichment in a specific cell subset. Indeed, expression-profiling studies of various human blood cell subsets have identified cell type specific miRNAs [37]. In addition, gender differences have been reported for blood composition in healthy individuals, revealing higher counts for B and T lymphocytes in women, and higher counts for monocytes and NK cells in men [38,39]. Finally, frequencies of specific cell subsets are altered in RA, including higher counts for CD14^+^CD16^+^ monocytes, Th17 and B lymphocytes. Although future studies using purified cell subsets from male and female RA patients will be necessary to fully address this possibility, we performed an in silico analysis using data sets from Allantaz and coworkers (GSE28487) to extract the relative expression level of the X-linked miRNAs that we studied in various immune cell subsets including T and B lymphocytes, NK cells and monocytes (Figure S5). Results showed that, out of the 14 X-linked miRNAs studied, eight are similarly expressed in the four immune cell types analyzed and three are preferentially expressed in monocytes (miR-188, miR-223 and miR-532).

Studies of expression quantitative trait loci (eQTL) offer promise for understanding gene regulation through genetic variants that might explain variations in gene expression levels. Here, we have identified two miRNA clusters neighboring genetic variants that have been previously associated with RA and localized in the *FOXP3* gene. These two *FOXP3* SNPs (rs3761548 and rs223236) are localized in the promoter region and are correlated with disease activity, joint damage, and laboratory variables [40]. Such associations suggest that T cells take part in the pathogenesis of RA. In the present study, we identified a significant miR-eQTL for both *FOXP3* polymorphisms between FoxP3 polymorphism rs3761548A/C and miR-221, miR-222, and miR-532 expression levels, as well as between *FOXP3* polymorphism rs2232365A/G and miR-221 expression level, only when stratifying by gender. Our data suggest that SNP genetic alterations of miRNA expression are strictly gender-dependent. Recently, Kukurba et al. evaluated the effect of both sex and genetic variation on patterns of gene expression, comparing X chromosome and autosomes [41]. Using whole blood transcriptomes, they demonstrated that genes on the X chromosome are more likely to have sex-specific expression compared to genes on the autosomes, and found an enrichment of sex-interacting eQTLs on the X chromosome. They identified that a portion of this difference was due to the hemizygosity of the X chromosome in males where the exposure of individual alleles is not balanced by random X-inactivation. In addition, these authors showed that these genes were more likely to be involved in apoptosis and regulation of cell death, which is consistent with divergent regulation of cell death programs between the sexes [42,43]. Here, we have identified three miRNA-eQTLs that might contribute to sex-biased disease risk in RA. Indeed, we found higher expression levels of miR-221, miR-222, and miR-532 in male than in female patients with RA harboring allele A of rs3761548. On the one hand, the rs3761548 is an intronic *FOXP3* polymorphism shown to be associated with autoimmune disease susceptibility and reduced transcription of the *FOXP3* gene [44,45]. *FOXP3* is a master regulator of the development and function of Treg cells [46,47], which play an essential role in preventing autoimmunity by contributing to the maintenance of immunological self-tolerance and immune homeostasis, and whose number and/or function is altered in RA [48]. On the other hand, miR-221, miR-222 and miR-532 control pro-inflammatory and pro-survival genes in different cellular context. More specifically, miR-221 controls T cell proliferation and survival by targeting three genes (PIK3R1, FOS and IRS2) [49]. Therefore, one can speculate that RA female patients harboring the allele A of rs3761548 in *FOXP3* will display defective Treg numbers and functions due to lower *FOXP3* transcription, which will be associated with higher T cell proliferation and inflammation due to lower miR-221, miR-222 and miR-532 expression levels. Although RA male patients harboring the allele A of rs3761548 in *FOXP3* will also display defective Treg numbers and functions due to lower *FOXP3* transcription, these Treg alterations will be counterbalanced by higher miR-221, miR-222 and miR-532 expression levels that will lead to reduced cell proliferation and inflammation compared to RA women. Overall, our results suggest that the Treg defect due to the rs3761548 polymorphism in *FOXP3* will be higher in RA females than in males because of associated miRNA-eQTLs.

Finally, differences in miRNA expression levels may be due to genetic differences but also to other factors such as decay processes. Indeed, previous studies have shown that mature miRNA expression level can be modulated without variation in the expression pattern of the precursor forms pri- and pre-miRNAs, supporting the existence of regulatory mechanisms acting on the mature miRNA stability [50]. In addition, stability between primary and mature miRNAs can vary, depending on their hairpin structure and length (100–150 to 20–25 nucleic acids, respectively) [51]. We thus monitored the transcription of the three pri-miRNAs encoding for mature miR-221, miR-222 and miR-532. Although not significant (probably due to the small size of samples), the differences of expression of these three pri-miRNAs display similar patterns to those observed with the expression of the mature miRNAs (Figure S6), suggesting that the observed differences in miRNA expression levels between the groups might be due to differences in transcription activity. However, other post-transcriptional processes cannot completely be ruled out and will need future investigation.

To the best of our knowledge, this is the first human study to reveal a sexual dimorphism in miRNA expression in RA disease, in particular for six X-linked miRNAs. In addition, we identified miRNA-eQTLs with two SNPs localized within the *FOXP3* gene that might yield insights into regulatory mechanisms of variations in miRNAs transcription. Our study suggests that exploring X-linked miRNAs in the context of sex differences may provide novel insight into the pathophysiology of RA and autoimmunity, and identify potential therapeutic targets for treatment and prevention.

## 4. Materials and Methods

### 4.1. Human Subjects

For RA patients (*n* = 21), informed consents were provided in accordance with national procedures. French studies were approved by local human ethical committees: Sample collection and analysis (DC-2008-327) was approved by the “Cellule Bioéthique, direction générale pour la recherche et l’innovation, Ministère de l’Enseignement Supérieur et de la Recherche” (Ministry bioethics unit) and by Comité de Protection des Personnes Sud Méditerrannée IV (ID RCB 2008-A01087-48). All RA patients fulfilled the 2010 ACR/EULAR classification criteria. RA patients presented a low disease activity (DAS28 < 2.6) with over 76% of patients in remission. Equal number of RA males and females were prospectively recruited. Twenty-two age- and sex-matched healthy controls were recruited from the Etablissement Français Du Sang (EFS) of Montpellier (Table 1).

### 4.2. Preparation of Blood Samples

Blood samples were collected with EDTA-2K containing tubes: 2 tubes for total RNA extraction including miRNAs and one tube for DNA extraction. For miRNAs extraction, mononuclear cells from peripheral blood were separated by ficoll-hypaque density gradient (GE Healthcare, Buckinghamshire, UK). Total RNAs including small RNAs were extracted using miRNeasy Mini Kit with a Qiacube (QIAGEN) according to the manufacturer’s instructions. RNA concentrations were assessed using the NanoDropTM spectrophotometer (Thermo Fisher Scientific, Waltham, MA, USA). Genomic DNA was extracted using QIAamp DNA Blood Maxi kit (catalog No51104, QIAGEN, Hilden, Germany) according to the manufacturer’s instructions.

### 4.3. Quantification of Mature and Pri-miRNAs

Total RNAs (35 ng) were converted into cDNAs using Multiplex RT Primers that contain a pool of 13 individual miRNA-specific primers (hsa-miR-146a, hsa-miR-223, hsa-miR-363, hsa-miR-106a, hsa-miR-20b, hsa-miR-188, hsa-miR-92a, hsa-miR-532, hsa-miR-652, hsa-miR-221, hsa-miR-222, hsa-miR-98, let-7f and endogenous control RNU48) and TaqMan MicroRNA Reverse Transcription kit, following a pre-amplification using homemade MegaplexTM PreAmp Primers and TaqMan^®^ PreAmp Master Mix, according to the manufacturer’s instruction. TaqMan^®^ Advanced Master Mix was used for quantitative PCR and reaction performed on a Viia7 real-time PCR system (Applied Life Technologies, Saint Aubin, France). All reagents were supplied by Life Technologies (Saint Aubin, France). The endogenous control RNU48 was used for data normalization. Expression levels of pri-miRNAs encoding for mature miR-221, miR-222 and miR-532 were quantified using pri-miRNA Gene expression assays (Life Technologies), following the manufacturer’s instructions. The expression GAPDH was used as endogenous control. Relative miRNA and pri-miRNA expressions were calculated using the comparative threshold cycle (*C*_t_) method.

### 4.4. SNPs Identification

All subjects were genotyped for the two FoxP3 polymorphisms rs3761548 and rs2232365 using direct PCR sequencing with the BigDye Terminator v3.1Cycle Sequencing Kit (Applied Biosystems) and an Applied Biosystems (ABI) 3130xL genetic analyzer (Applied BioSystems, Foster City, CA, USA). PCR reactions were performed on a Bio-Rad thermal cycler using Taq Polymerase (Prim 5 by Fisher Scientific) with initial denaturing conditions at 96 °C for 5 min, followed by 30 cycles of 96 °C for 30 s, 64 °C for 30 s, 72 °C for 45 s, and a final extension of 72 °C for 10 min.

### 4.5. Statistical Analysis

Data are presented as the mean ± standard deviation. Statistical analyses were performed using GraphPad prism software (version 6). Correlations between miRNAs expression, clinic pathological parameters and FoxP3 genotypes were analyzed with Pearson r test and Mann–Whitney test, respectively. For quantitative variables, One-way ANOVA test and Mann–Whitney test were used for parametric and non-parametric variables, respectively. Chi-square test was used for quantitative variables. Probability values less than 0.05 were considered significant.

## 5. Conclusions

The aim of the present study was to determine whether expression levels of some X chromosome-linked miRNAs were different between RA patients and healthy donors as well as between males and females. Our analysis highlighted sex differences of the expression of several X chromosome-linked miRNAs. Interestingly, eQTL study showed that two FoxP3 polymorphisms associated with RA susceptibility may influence sex-biased miR-221 expression levels.

## Figures and Tables

**Table 1:** Characteristics of control subjects and patients with rheumatoid arthritis.

Characteristics	RA	HC
Number of samples	21	22
Sex, male/female (% women)	10/11 (52.4)	11/11 (50)
Age, mean ± SD (years)	60 ± 12.0	54.7 ± 6.4
Disease duration (years)	16.1 ± 13.3	NA
Positive ACPA, *n* (%)	21 (100)	NA
Positive RF, *n* (%)	21 (100)	NA
C-reactive protein (mg/L)	14.1 ± 23.2	NA
DAS28	2.6 ± 1.4	NA
ESR	21.8 ± 18.7	NA
Drug use, *n* (%)		
Infliximab	7 (41.2%)	NA
Tocilizumab	5 (29.5%)	NA
Rituximab	5 (29.5%)	NA
Adalimumab	1 (5.4%)	NA
Prednisolone	5 (29.5%)	NA
Methotrexate	7 (41.2%)	NA

Values are expressed as the mean (±standard errors) for continuous variables or as percentages for categorical variables. Abbreviations: NA: not applicable; RA: Rheumatoid arthritis; HC: healthy controls; ACPA: Antibodies to citrullinated protein antigen; RF: Rheumatoid factor; DAS28: Disease Activity Score-28; ESR: Erythrocyte sedimentation rate.

**Table 2:** X-linked miRNAs associated with RA polymorphisms.

miRBase ID	Chromosome	Position (pb)	Gene	Location miRNA/Gene	Distance miRNA-Gene	RA SNPs	MAF
miR-221	Xp11.35	45746157–45746266	*Foxp3*	Intergenic	≈3.5 Mb	rs3761548, rs2232365	0.24, 0.41
miR-222	Xp11.3	45747015–45747124	*Foxp3*	Intergenic	≈3.5 Mb	rs3761548, rs2232365	0.24, 0.41
miR-532	Xp11.2	50003148–50003238	*CLCN5*	Intragenic	-	NF	NF
			*Foxp3*	Intergenic	≈733 Kb	rs3761548, rs2232365	0.24, 0.41
miR-188	Xp11.2	50003503–50003588	*CLCN5*	Intragenic Intergenic	-	NF	NF
			*Foxp3*		≈500 Kb	rs3761548, rs2232365	0.24, 0.41
miR-98	Xp11.2	53556223–53556341	*HUWE1*	Intragenic	-	NF	NF
let-7f-2	Xp11.2	53557192–53557274	*HUWE1*	Intragenic	-	NF	NF
miR-223	Xq12	66018870–66018979	*VSIG4*	Intergenic	≈2868 bp	NF	NF
miR-652	Xq23	110055329–110055426	*TMEM164*	Intragenic	-	NF	NF
miR-363	Xq26	134169378–134169452	Non coding gene	-	-	NF	NF
miR-92a-2	Xq26	134169538–134169612	Non coding gene	-	-	NF	NF
miR-20b	Xq26	134169809–134169877	Non coding gene	-	-	NF	NF
miR-106a	Xq26	134169809–134169877	Non coding gene	-	-	NF	NF
miR-3202	Xq28	154019920–154019989	TMEM187	Intragenic	-	rs17422	0.41
miR-718	Xq28	154019920–154019989	IRAK1	Intragenic	-	rs1059702, rs1059703, rs1734792	0.37, 0.48, 0.42

Abbreviations: RA: Rheumatoid arthritis; SNP: single nucleotide polymorphism; MAF: Minor allele frequency; FoxP3: forkhead box P3; CLCN5: chloride voltage-gated channel 5; HUWE1: HECT; UBA and WWE domain containing 1; VSIG4: V-set and immunoglobulin domain containing 4; TMEM164: transmembrane protein 164; IRAK1: interleukin 1 receptor associated kinase 1; MECP2: methyl-CpG binding protein 2; TMEM187: transmembrane protein 187; NF: Not found.

**Table 3:** Correlation between X-linked miRNAs expression and RA biological parameters.

	DAS28	Anti-CCP	CRP	RF	Disease Duration	ESR
**miR-221**	*p = 0.510*	*p = 0.151*	*p = 0.520*	*p = 0.629*	*p = 0.474*	***p = 0.005*, r = 0.65**
**miR-222**	*p = 0.255*	*p = 0.246*	*p = 0.267*	*p = 0.280*	*p = 0.800*	***p = 0.004*, r = 0.66**
**miR-532**	*p = 0.484*	*p = 0.312*	*p = 0.510*	*p = 0.723*	*p = 0.609*	*p = 0.687*
**miR-188**	*p = 0.910*	*p = 0.866*	*p = 0.534*	*p = 0.971*	*p = 0.288*	*p = 0.979*
**miR-98**	*p = 0.388*	*p = 0.242*	*p = 0.342*	*p = 0.332*	*p = 0.447*	*p = 0.055*
**let-7f-2**	*p = 0.501*	*p = 0.261*	*p = 0.338*	*p = 0.176*	*p = 0.204*	***p = 0.007*, r = 0.62**
**miR-223**	*p = 0.659*	***p = 0.004*, r = 0.63**	*p = 0.423*	*p = 0.905*	*p = 0.551*	*p = 0.475*
**miR-652**	*p = 0.694*	*p = 0.190*	*p = 0.753*	*p = 0.662*	*p = 0.775*	***p = 0.036*, r = 0.51**
**miR-363**	*p = 0.740*	*p = 0.223*	*p = 0.473*	*p = 0.731*	*p = 0.164*	*p = 0.737*
**miR-92a-2**	*p = 0.272*	*p = 0.201*	*p = 0.319*	*p = 0.131*	*p = 0.287*	*p = 0.057*
**miR-106a**	*p = 0.147*	*p = 0.326*	*p = 0.283*	*p = 0.201*	*p = 0.111*	*p = 0.283*
**miR-20a**	*p = 0.790*	*p = 0.081*	*p = 0.909*	*p = 0.219*	*p = 0.081*	*p = 0.909*

Abbreviations: RA: Rheumatoid arthritis; DAS28: Disease Activity Score-28; Anti-CCP: Antibodies to citrullinated protein antigen; CRP: C-reactive protein; RF: Rheumatoid factor; ESR: Erythrocyte sedimentation rate. Significant correlations are indicated in bold (*p*-value and r).

## Supplementary Materials

Supplementary materials can be found at www.mdpi.com/1422-0067/17/11/1852/s1.
