# Supplementary material for: X-Linked miRNAs Associated with Gender Differences in Rheumatoid Arthritis
Source: Int J Mol Sci. 2016 Nov 8;17(11):1852. doi: 10.3390/ijms17111852 (PMC5133852; doi:10.3390/ijms17111852)
Supplement: Supplementary file 1 [file ijms-17-01852-s001.pdf]

Supplementary Materials: X-Linked miRNAs Associated with Gender Differences in Rheumatoid Arthritis

Olfa Khalifa, Yves-Marie Pers, Rosanna Ferreira, Audrey Sénéchal, Christian Jorgensen, Florence Apparailly and Isabelle Duroux-Richard

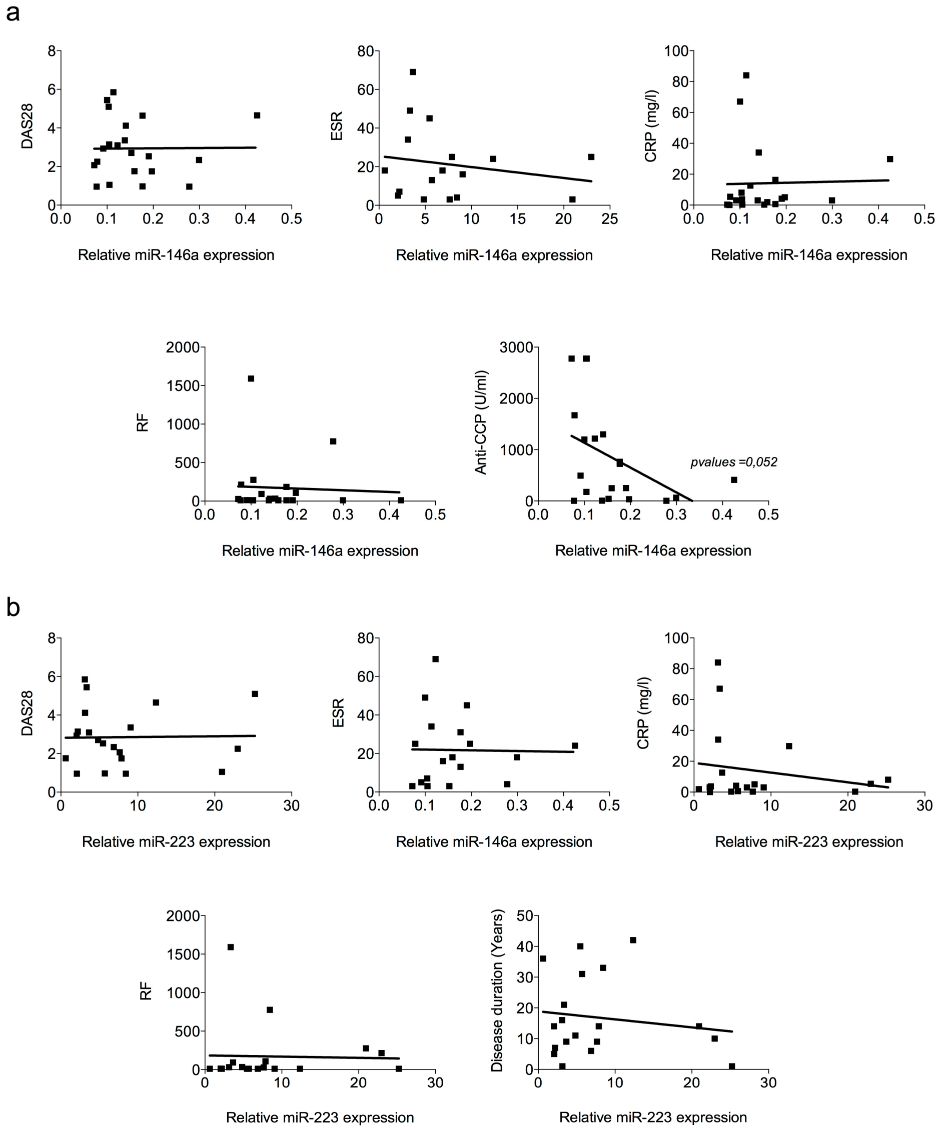

Figure S1. Absence of correlation between the expression levels of miR-146a and miR-223 and biologic parameters of patients with non-active RA disease. Expression levels of X-linked miRNAs in PBMCs from RA patients and healthy subjects were quantified using RT-PCR, and endogenous RNU48 was used for normalization**.** Correlation between miR-146a (**a**) and miR-223 (**b**) expression levels and 5 biological parameters (DAS28, ESR, CRP, FR Anti-CCP and Disease duration) are shown. Abbreviations: RF: Rheumatoid factor, DAS28: Disease Activity Score-28, CRP: C-reactive protein, anti-CCP: Antibodies to cyclic citrullinated peptides, and ESR: Erythrocyte sedimentation rate.

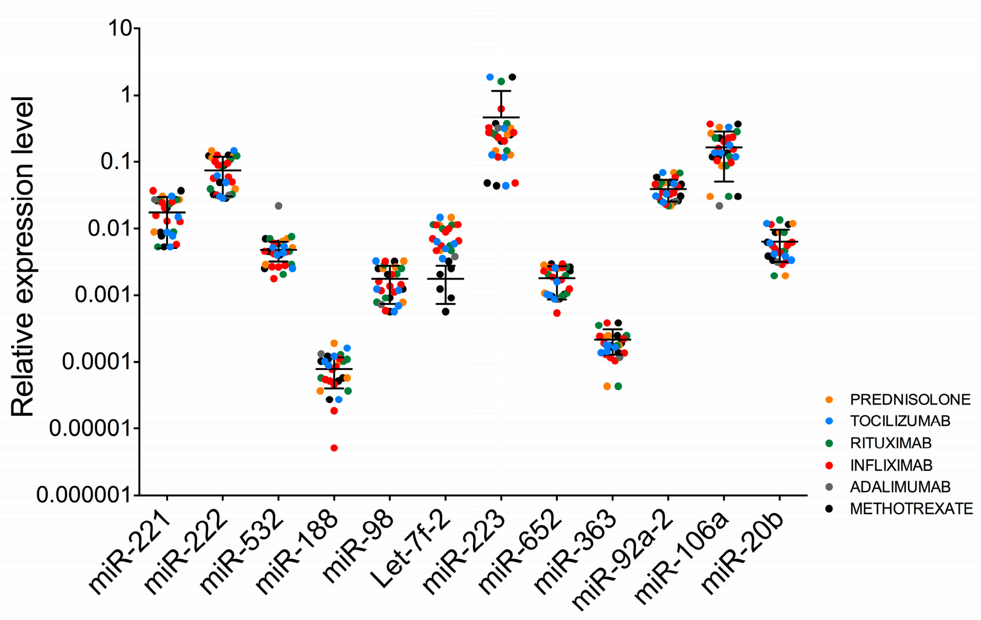

Figure S2. Treatment does not influence the expression levels of X-linked miRNAs in RA patients. Expression levels of X-linked miRNAs were detected on PBMCs of RA patients (*n* = 21) using RT-PCR, and endogenous RNU48 was used for normalization. Data are plotted according to the treatment: Tocilizumab (**blue circles**), Rituximab (**green circles**), Infliximab (**red circles**), Adalimunab (**grey circles**), Prednisolone (**orange circles**) and Methotrexate (**black circles**).

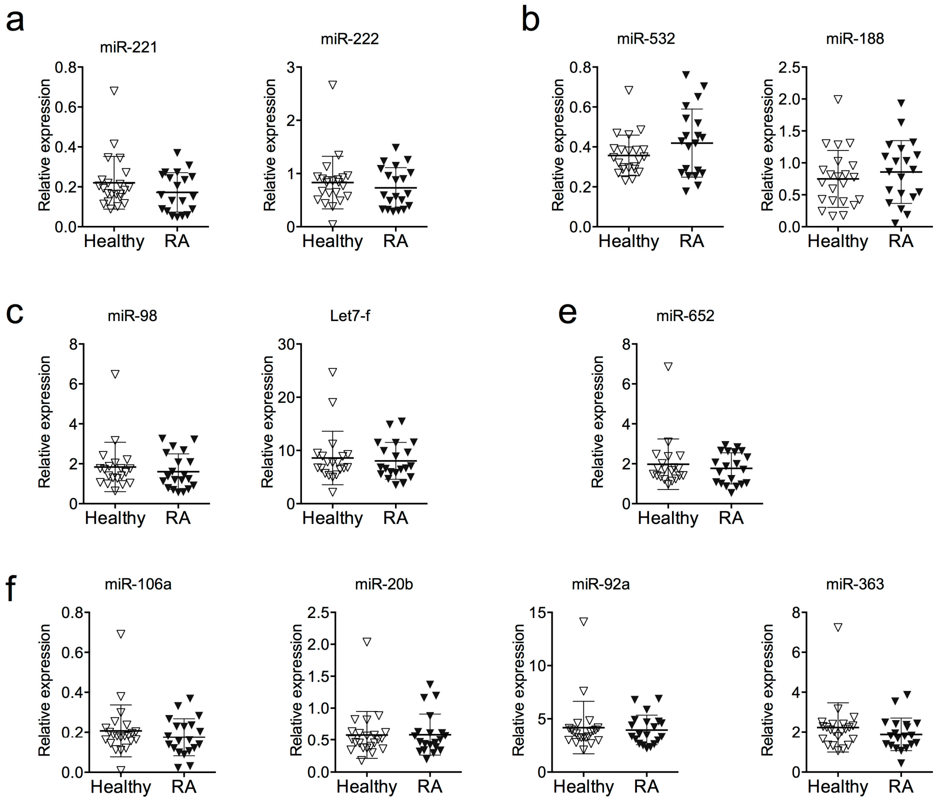

Figure S3. Comparison of X-linked miRNAs expression levels in RA and healthy subjects PBMC samples without gender stratification. Expression levels of X-linked miRNAs were detected on PBMCs of RA patients and healthy subjects using RT-PCR, and endogenous RNU48 was used for normalization**.** Expression levels of miR-221 and miR-222 (**a**); miR-532 and miR-188 (**b**); miR-98 and Let7-f (**c**); miR-652 (**e**) miR-106a, miR-20d, miR-92a and miR363 (**f**) are plotted without gender stratification. Results are expressed as mean ± SD of individual samples of 21 RA patients and 21 healthy subjects.

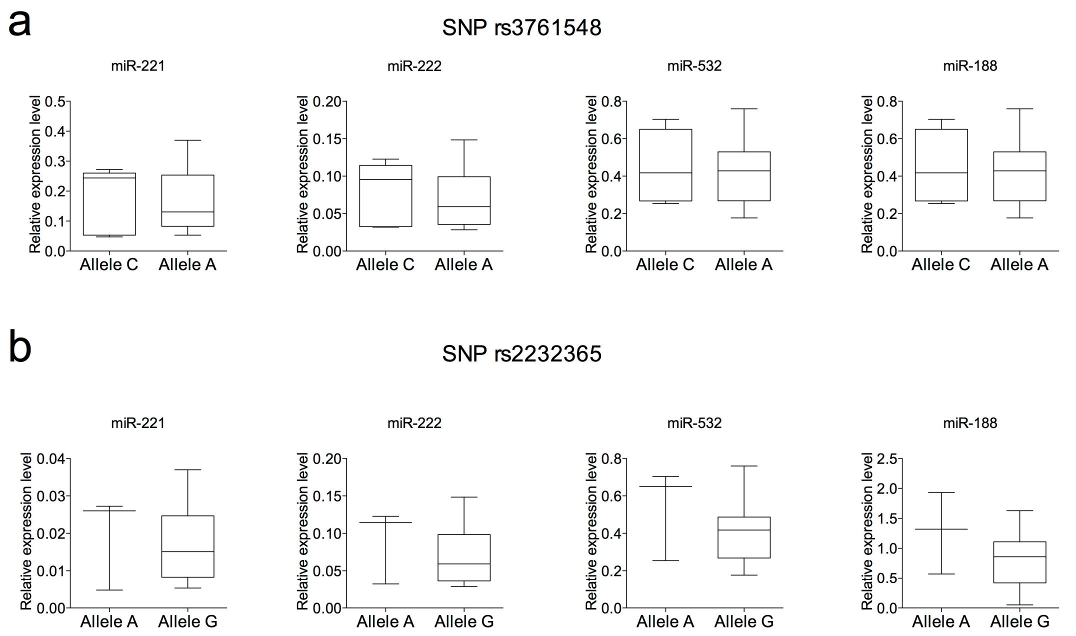

Figure S4. Expression levels of 4 miRNAs located near 2 *FOXP3* polymorphisms associated with RA susceptibility without gender stratification. To assess the relationship between genetic variation and the expression level of X-linked miR-221/222 and miR532/188 clusters miR-221, miR-222, miR-532 and miR-188 expression levels were quantified in RA patients using RT-PCR and DNA from RA patients was genotyped using direct PCR sequencing with the BigDye Terminator v3.1Cycle Sequencing Kit. Data are presented according to the *FOXP3* genotype, rs3761548 (**a**) and rs2232365 (**b**) and to the mutated versus wild type genotype. Box plots are the representation of individual sample of 21 RA patients.

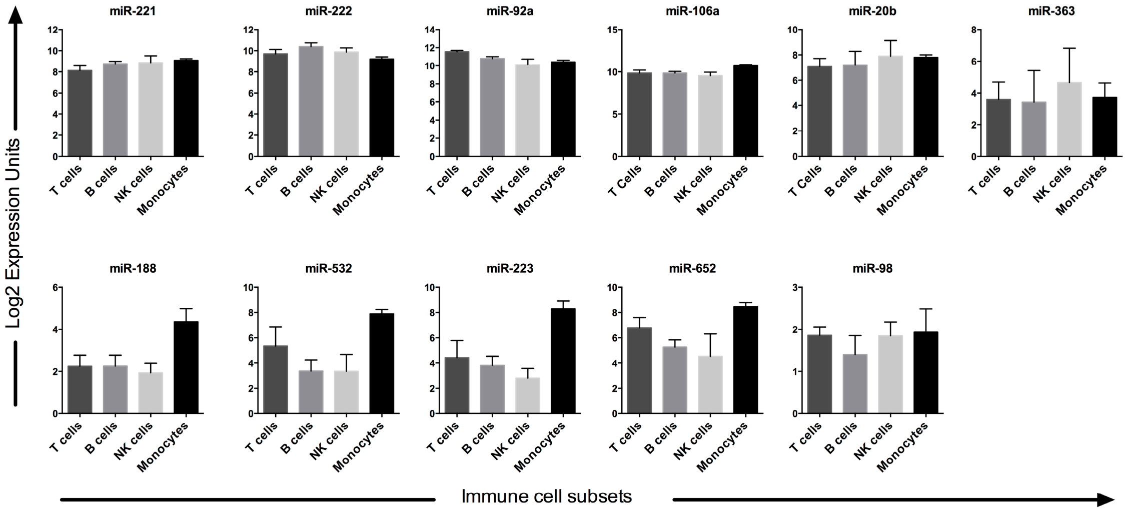

Figure S5. Cell type-specific expression of X-linked miRNAs in healthy donors. The expression levels of 11 X-linked miRNAs in different immune cell types were extracted from the GSE28487 data sets. Eight X-linked miRNAs (miR-221, miR-222, miR-92a, miR-106a, miR-20b, miR-363, miR-98 and miR-652) are similarly expressed between all human immune cells types investigated and 3 miRNAs (miR-188, miR-532, and miR-223) appear to be preferentially expressed in monocytes. Relative miRNA expressions are represented as log2 signal value.

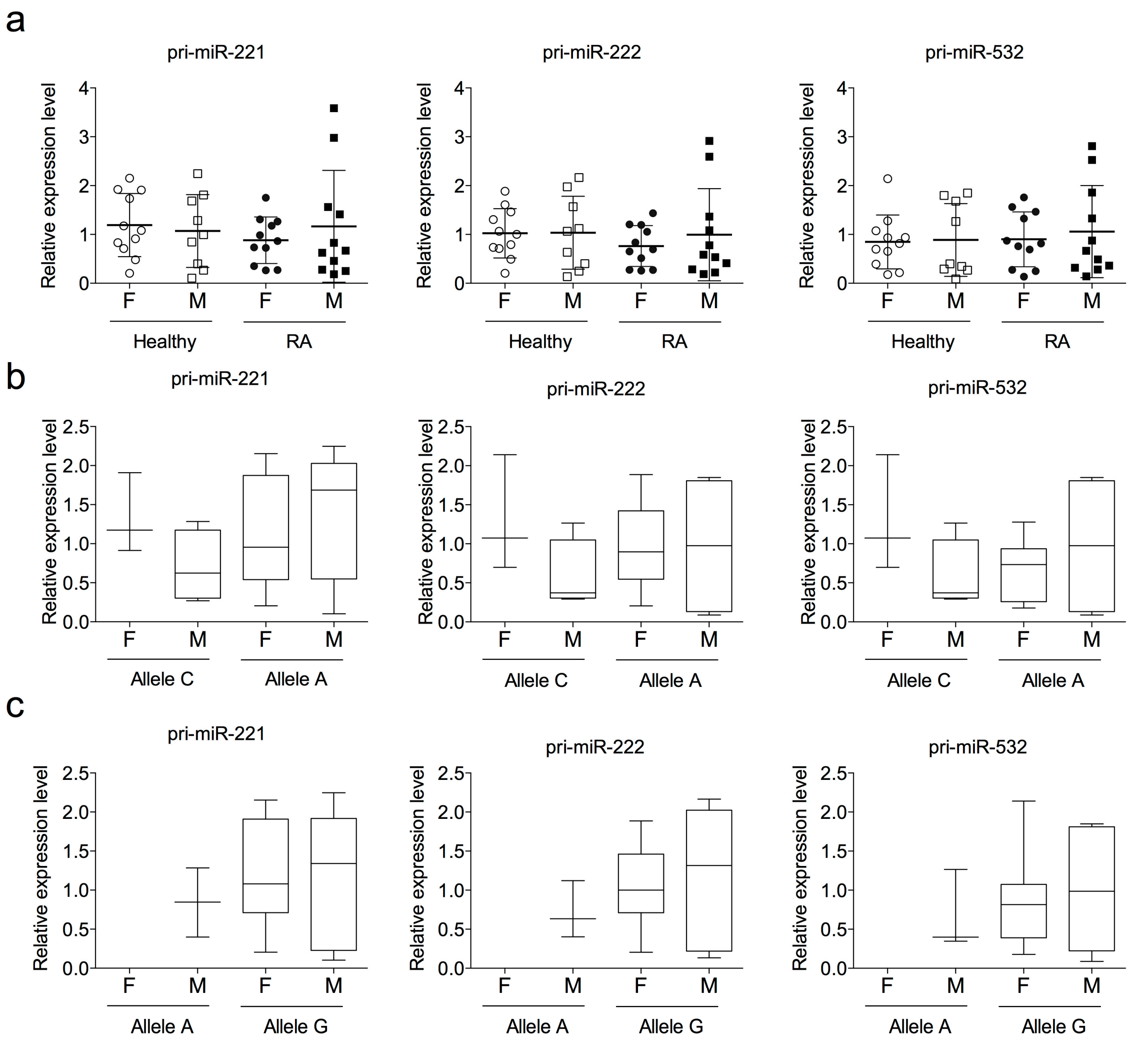

Figure S6. Expression levels of X-linked miRNA precursors in RA patients according to gender stratification and *FOXP3* genotypes. The expression level of X-linked miR-221/222 and miR532/188 cluster precursors were quantified using RT-PCR in RA patients (*n* = 21) and healthy controls (*n* = 22) and stratified according to the gender (**a**); The relationship between genetic variation in the *FOXP3* promoter and miRNA precursors are presented (**b**,**c**). DNA from RA patients was genotyped using direct PCR sequencing with the BigDye Terminator v3.1Cycle Sequencing Kit. Data are presented according to the *FOXP3* genotype: rs3761548 (**b**) and rs2232365 (**c**) and to the mutated versus wild type genotypes.
